# The preschool strengths inventory: development and validation

**DOI:** 10.3389/fpsyg.2025.1468944

**Published:** 2025-02-12

**Authors:** Rhea L. Owens, Meagan M. Patterson, Karen D. Multon

**Affiliations:** ^1^Department of Psychology, University of Minnesota Duluth, Duluth, MN, United States; ^2^Department of Educational Psychology, University of Kansas, Lawrence, KS, United States

**Keywords:** strengths, assets, early childhood, assessment, positive psychology, measure, instrument

## Abstract

There is a lack of research on young children’s strengths, likely in part due to limited tools available to identify individual strengths in early childhood. To help address this gap and provide a brief measure for parents, researchers, and practitioners, the 37-item *Preschool Strengths Inventory* (PSI) was developed. First, focus groups with parents were conducted to identify strengths in early childhood. Based on the results of the focus groups and a review of the research, items were developed, reviewed by experts, and tested through exploratory and confirmatory factor analysis. The five factors identified were: *Dynamic, Dependable, Caring, Inspiring,* and *Organized.* Lastly, validity was tested and established with measures of personality traits and social skills, and the PSI’s test–retest reliability was examined. Results provide support for the content structure, reliability, and validity of the PSI. The PSI can provide the ability to study strengths beginning early in life and provide a foundation to develop strengths-focused interventions.

## Introduction

Strengths are central to the field of positive psychology and the strengths literature has established that identifying, using, and developing strengths can ameliorate risk factors and promote well-being (e.g., Ghielen et al., 2018; Lavy, 2019; Louis and Lopez, 2014; Seligman and Csikszentmihalyi, 2000). Although the benefits of strengths are apparent across a variety of contexts, the literature related to strengths has focused primarily on adults and adolescents, with limited research involving children (Owens and Waters, 2020; Proctor et al., 2011; Quinlan, 2015). Despite the increasing attention to positive psychology principles in early childhood development and education in recent years (e.g., Baker et al., 2017; Lottman et al., 2017; Waters et al., 2022), there has been relatively little focus on the identification of individual-level strengths in young children. Therefore, the purpose of the studies presented in this paper was to develop and validate a brief strengths measure to identify individual, internal strengths present in children 3–5 years of age.

From a prevention and early intervention perspective, focusing attention on the strengths development process early in childhood is advantageous (Galloway et al., 2020; Hage et al., 2007; Nelson et al., 2003; Owens et al., 2018; Owens and Waters, 2020). The strengths development process begins with the identification of strengths (Clifton et al., 2006). Thus, assessments with the goal of identifying individual strengths in young children can help provide the means to begin the strengths development process early in life, likely resulting in a number of beneficial outcomes. For example, a developmentally appropriate framework for reflection on children’s strengths may help parents to provide appropriate forms of praise (e.g., non-inflated praise; Brummelman et al., 2017) or facilitate parent–child conversations about strengths, which can in turn promote positive parent–child relationships (Goodman et al., 2021). In the school setting, teachers could help students learn to use their individual strengths to enhance their educational experience and learning outcomes (Galloway et al., 2020). Related to mental health, developmentally appropriate strengths interventions could be designed to enhance well-being and address challenges, a noticeable gap in the literature (Owens and Waters, 2020).

### Strengths in early childhood

In the positive psychology literature, there are several conceptualizations and definitions of strengths (Louis and Lopez, 2014). Across definitions, strengths are generally viewed as unique dispositional characteristics that represent what people are good at and promote positive outcomes, such as well-being (Biswas-Diener et al., 2011; Louis and Lopez, 2014). The definition that guided the development of the PSI extends from the trait theory framework (Carr, 2004): strengths represent positive traits or skills that promote optimal functioning (Owens et al., 2018). In line with this framework, strengths are observed to be fairly stable across the lifespan, but they are not fixed and can be strengthened as a result of environmental/contextual factors or interventions (e.g., Biswas-Diener et al., 2011; Bowers et al., 2010; Ghielen et al., 2018; Owens et al., 2018). Strengths can also be characterized into higher-order and lower-order characteristics, similar to the classification of personality traits (Caspi and Shiner, 2006; Putnam et al., 2001).

Although there are commonalities in the conceptualization of strengths with personality traits and key developmental tasks/abilities, there are some important distinctions. Personality traits represent stable ways in which people differ from one another without valence or the goal of achieving desirable outcomes (Funder, 2024). Developmental tasks tend to reflect what is necessary or expected for children of a given age or developmental stage (McCormick et al., 2011). Conversely, strengths are valenced (i.e., focused on positive characteristics resulting in beneficial outcomes) and reflect characteristics that are particularly strong within an individual compared to their peers or other characteristics and abilities they possess (Biswas-Diener et al., 2011; Louis and Lopez, 2014). Existing conceptualizations of strengths also tend to focus on traits, rather than cognitive developmental factors such as intelligence, memory, or executive functioning or specific abilities and talents such as athletic or musical ability (Biswas-Diener et al., 2011; Louis and Lopez, 2014; Shoshani, 2019). For young children in particular, strengths may be incorporated into identity in a way that personality traits and developmental tasks are not, given young children’s tendencies to focus on accomplishments and positive characteristics when thinking about the self (Harter, 1999).

As noted above, there is little research on strengths in early childhood that focuses on identifying and measuring strengths specific to this developmental stage. Our approach to strengths identification incorporates both inductive (i.e., focus groups) and deductive (i.e., review of developmental literature) methods for item generation. Although inductive methods relying on the qualitative responses of parents provided the overall structure for measurement, we expected that some of the strengths identified from the focus groups would likely map onto established constructs from research in early childhood development. For instance, the strength of kindness might overlap with elements of social competence, and the strength of positivity might overlap with the positive emotionality dimension of temperament. Our focus on a trait-like conceptualization of strengths connects to the literature on early childhood temperament and personality (Caspi et al., 2005; Shiner and DeYoung, 2013; Slobodskaya, 2021) and aligns with the individual strengths literature (Carr, 2004; Louis and Lopez, 2014).

### Existing strengths measures

Although a number of strengths measures are available for adults (e.g., Lopez et al., 2005; Peterson and Seligman, 2004) and adolescents (e.g., Leffert et al., 1998; Lopez et al., 2005; Park and Peterson, 2006), few measures exist for assessing individual strengths in young children. To the authors’ knowledge, there are currently three extant measures of strengths for preschool-aged children: the Behavioral and Emotional Rating Scale (BERS-2; Buckley and Epstein, 2004; Epstein et al., 2009; ages 5–18), the Devereux Early Childhood Assessment (DECA; LeBuffe and Naglieri, 1999; ages 2–5), and the Character Strengths Inventory for Early Childhood (CSI-EC; Shoshani, 2019; ages 3–6). However, neither the BERS-2 nor the DECA focus on *individual, internal* strengths of young children from a promotive perspective (i.e., designed to focus on positive characteristics without the presence of problems or risk). Rather, both the BERS-2 and DECA were designed based upon resilience literature. Despite the importance of resilience as a construct, it implies that some form of risk or barrier is present (Zolkoski and Bullock, 2012).

The CSI-EC is a parent report measure that was developed to measure young children’s strengths consistent with the VIA classification of character strengths (Shoshani, 2019). The measure includes 24 strengths clustered into four areas (i.e., interpersonal, intellectual, temperance, and transcendence). Interpersonal strengths include social intelligence, love, kindness, perspective, leadership, bravery, fairness, and teamwork. Intellectual strengths include love of learning, curiosity, appreciation of beauty, and creativity. Temperance strengths include self-regulation, modesty, prudence, forgiveness, open-mindedness, and persistence. Transcendence strengths include humor, zest, hope, authenticity, spirituality, and gratitude. Although the items of the CSI-EC were designed to assess parents’ perceptions of strength in children ages 3–6, the measure was created by writing items to align with the existing 24 character strengths of the VIA classification—strengths that were originally identified with an adult sample. The goal of this study and the PSI was to create a tool specifically designed to identify individual, internal strengths in early childhood.

### The present research

Identifying and developing strengths has many benefits for individuals across the lifespan; however, those benefits are scarcely represented in the research on young children. Assessment tools with a focus on internal, individual strengths could help further the literature and establish evidenced-based strengths practices for young children.

To develop the PSI, several steps were taken. First, focus groups with parents of young children (ages 3–5) were conducted to identify specific strengths (Study 1). Scale items were created based on data from the focus groups as well as constructs from existing developmental literature, and an exploratory factor analysis (EFA) followed by a confirmatory factor analysis (CFA) were conducted of the items generated (Study 2). Finally, test–retest reliability was examined and validity was tested with measures of personality traits and social skills (Study 3).

## Study 1: parent focus groups

In study 1, focus groups with parents of preschool-aged children were conducted to inform the development of items for the PSI.

### Methods

#### Participants

Parents/guardians of 3–5 year old children (*M* = 3.60, *SD* = 0.87) were recruited through preschools and day care centers in the Midwestern United States. Participation was voluntary and no compensation was provided to participants. Seventeen parents (65% mothers, 35% fathers, age *M* = 36.94, *SD* = 6.27) participated in one of four focus groups. The parent sample was 70% White, 12% Asian, 6% African American/Black, and 12% of another race/ethnicity.

#### Procedure

A semi-structured interview protocol was developed by the first author and reviewed by several experts in developmental, school, and counseling psychology. The questions were designed to facilitate discussion about what strengths parents recognized in children across different settings to help identify which strengths to include in the initial item set. Questions included: (a) What is your definition of a strength? (b) Tell me about a time your child was at their best. What did that look like in your child? (c) What are your child’s strengths? What is your child particularly good at? At what age did your child begin showing these strengths? (d) What positive behaviors do you see in your child in different settings (home, school, with friends, with family, alone, etc.)? (e) What positive behaviors do you see in other children that you do not necessarily see in your child? The focus groups were conducted by the first author and were 45–75 min in length.

The focus groups were audio-recorded and later transcribed by the first author and two trained research assistants. The data was analyzed and coded using a basic interpretative approach (Merriam and Grenier, 2019). This method was chosen to discover and understand the perspectives of parents’ perceptions of young children’s strengths. With this method, data are inductively analyzed to identify common themes across the data.

When coding, the researchers listened for and kept an ongoing list of adjectives or traits either described or explicitly labeled by the parents and independently generated a list of characteristics. These characteristics were then organized into broader categories by the researchers based on similarities among the characteristics. Both the individual characteristics and broader themes were compared and discussed among the three researchers until a consensus within the group was reached.

Several qualitative best practice strategies were used throughout the research process (see Brod et al., 2009; Johnson et al., 2020; Whittemore et al., 2001). These included purposive sampling of young children’s parents, using a semi-structured interview, and one researcher leading all of the focus groups. The focus groups were also conducted until it appeared data saturation was reached; saturation was judged as being met when there was repetition of themes across the focus groups and no new additional strengths were identified. In addition, the focus groups were transcribed verbatim, and prior to starting the coding, the researchers engaged in researcher reflexivity by acknowledging and discussing their potential biases with one another. During the coding process, researcher triangulation was achieved by involving three researchers, and early on a literature review was conducted to see how emerging themes aligned with the existing literature. Finally, an auditor and expert checking were used (e.g., Brod et al., 2009; Johnson et al., 2020; Whittemore et al., 2001).

### Study 1 results and discussion

Four broad, overarching themes were identified from the parent focus groups. These included interpersonal strengths (i.e., characteristics that involve interacting with others), intrapersonal strengths (i.e., characteristics within a person), cognitive strengths, and physical strengths. Given the PSI’s goal to measure trait-like strengths, some cognitive strengths (e.g., memory) and physical strengths (e.g., coordination) mentioned in the focus groups were not included in the measure development process, as these abilities were outside of the scope of the intended measure. Examples of the themes identified from the focus groups can be found in Table 1. Overall, these themes are aligned with the theoretical basis of trait theory (Caspi and Shiner, 2006), which suggests there are a number of defined positive characteristics that represent individual differences across people.

**Table 1:** Example strengths from focus groups.

Strength	Parent examples
Active	“It’s really amazing that [spatial abilities] carries over to her physical abilities. She learned to do the monkey bars really early and she can do lots of things with her body and space that a lot of kids her age cannot do.” “My daughter is at her best during sports. When she’s doing what she knows. She’s just like, ‘Dad, did you see me catch that?’”
Creative	“She’s very creative. She tells these – even when she was little – she started telling these stories. And she uses literary devices in them.” “My little guy has an active imagination…He can literally play in his room with his dinosaurs for 30 to 40 min before he comes downstairs and tells me the story of the dinosaurs and what’s going on because dinosaurs have families…”
Curious	“…she’s very observant and kind of taking in everything that’s she’s seeing going on around her.”
Deliberate	“We were making gingerbread houses yesterday. Some of the kids were done in like 10 min and he sat there for 45 min very strategically putting things down. For him, he’s thinking things through very much.” “You know the deep thought processes and being able to take a long amount of time to be able to complete things and complete them well.”
Empathic	“I can see my daughter come up and put her hand around her [sister] and pat her neck, ask her if she can get her a glass of water when she cannot even reach the faucet.” “And she really, she really cares. It really struck me when Sarah got pinched in the swing…April did not quite see how it happened, but she was so worried about Sarah that she…tried her level best to try to hurt herself with the swing. She was trying to pinch herself with it and finally she just pushed it as hard as she could and let it bonk her on the head because it was the only way for her to figure out how to show her empathy for Sarah being hurt.”
Enthusiastic	“…very effervescent…she always loves to know what her friends are doing and get involved.”
Flexible	“My son is very good at adapting to difficult situations, like surprisingly. We lived abroad last year for a semester. I thought this would be really difficult for him; he’s just gung-ho about everything. You know, eating different food and just wanting to go on three airplanes in one day.” “…sometimes he comes to school with me and he’s there for 10 h, and being able to kind of go with the flow and not get upset easily…It’s just amazing to me he switches classrooms and he has different teachers in the morning than in the afternoon and he does not seem affected by it.”
Focused	“She’s really focused on what she’s doing when she’s doing it. And I’ve noticed that from her ever since she was a baby, so was always focused in on something and even to this day, her favorite thing is stuffed animals and she has this imaginary world with all her stuffed animals and she’ll be in her room for like an hour in her own little imaginary world and it’s very intricate, she very detailed-oriented…I’d say that’s her strength – to be very focused” “She got a sticker on her shirt because she was the only one paying attention. So I’m seeing things happening because she’s getting better and better at this. And that’s what’s kind of brought it out as one of her strengths is that she’s beginning to get recognized for it…She’s very focused in her room. She’ll just go in her room, and I like giving her time to do whatever she wants and she’ll come out like 20 min later with a very detailed drawing, age appropriate.”
Generous	“She’s very caring and generous, and she makes use of that as well.” “I can think of times when my daughter, mainly my oldest daughter, wanted something that she [my youngest daughter] had or needed and chose instead to keep, where the youngest would share it on her own without any prompting from me…it definitely as a parent it was a time I was impressed with her and her thought process and how she thought it was more important for her older sister to have it especially since she was the youngest.”
Gregarious	“Kaden is a natural. She just lights up the room. She just immediately makes friends. She’s one of those people that people want to make friends with. She’s just fun, giggly, and ridiculous.”
Helpful	“Harper, at school, she really likes to help people.” “When my youngest son was born, my daughter really stepped up to be responsible and helpful…She was like ‘I’ll help do this and I’ll help do that.’ She still likes to play mother hen.”
Humorous	“Well, she’s got a very good sense of humor. She knows how to get other kids to laugh.” “It seems like the sense of humor started younger, but he showed that as a strength even as young as 9 or 10 months just as an understanding and trying to make people laugh. Even when we went to his pediatrician for his 9-month check up and he went ‘Oh you have your hands full with this one. He’s going to be a partier!’ Because he just likes to have a good time. He always wants to have a good time and always laughs. But I think that sense of humor started pretty young, and some of the other things later…”
Leadership	“Hannah does really well if you give her leadership responsibility. She seems to really enjoy having the job…If I ask her to take care of her little sister, she’ll look after her. Just have to give her responsibilities and brings out the big sister in her.” “She’s very bossy and proud at home.”
Open-minded	“…she knows they are different, but she does not see things as gender, as boys or girls, or color [race or ethnicity].”
Organized	“My daughter, Helena, likes to order things and organize things.” “And she really likes organizing things…The teacher says she does not like to nap, so during nap time she folded her blanket square by square by square so to make it smaller and then unfold it and do it again so she can make a square.”
Persistent	“…she’s so persistent…but she gets something in her head and she just cannot be moved until she’s finished what she’s got going on…” “…She was at the park and she was climbing this climbing wall that was made for much bigger kids and her sister did not see her do it…So Kelsey [her sister] said, ‘Hey, do it again so I can see.” So Emily was like ‘Okay!’ and she gets halfway up, I’m standing back, and she gets halfway up and she falls off…I came back over and put her back on the wall and she finished climbing it, and I could see her little muscles shaking with fatigue from the force of the fall. But she finished climbing it because that’s what she set out to do.”
Positive	“She always plays with everybody, always laughing, always smiling…”
Self-disciplined	“I think there was a situation at school where someone took a toy away from her. I was dropping her off or something. She just kind of sat there and looked at them and tried to calculate what just happened and how she should handle it.” “She has fears about doing things for the first time, but she just does it and says, ‘I’m just going to be very careful so I can do it, I can do it, I can do it Mommy!’”
Warm	“And she has a lovely caring nature. She’s very protective of her younger siblings.” “Yeah, they really care about people. Yeah, when there’s a birthday party she knows, oh what would they like, and most children at that age, if you say ‘What do they want for their birthday?’ most children will say what they want…And Tessa will say, ‘Oh they want such and such,’ and I know it’s not something Tessa’s in[to].”

All names included are pseudonyms.

Upon completion of the focus groups, the strengths identified by parents were compared to the temperament, personality, and trait literature, which guided the final decisions of which traits were included in the item-development phase, discussed further in Study 2. Within the broad categories of intrapersonal and interpersonal strengths, many characteristics identified by the parents aligned with personality traits such as extraversion, openness, agreeableness, and conscientiousness (Caspi and Shiner, 2006). For example, strengths aligned with the trait of extraversion included descriptors such as active, enthusiastic, positive, gregarious, and leadership; strengths aligned with the trait of conscientiousness included deliberate, focused, organized, persistent, and self-disciplined. However, there were also some strengths that did not directly align with trait categories identified by Caspi and Shiner (2006), such as humorous.

## Study 2: instrument development

The purpose of study 2 was to build on the themes identified in study 1 to construct a quantitative measure of strengths that could be completed by parents to identify strengths in their young children. Steps for scale construction recommended by Walsh and Betz (2001) were used to guide the development of the PSI. First, a large pool of items was created and reviewed by experts. The items were then administered to an appropriate sample of participants. Finally, reliability estimates were examined.

### Study 2 methods

#### Scale construction

##### Item construction

The themes identified in study 1 were consistent with a trait-based approach to examining strengths in early childhood. We built upon these identified areas of strengths by incorporating related constructs from Caspi and Shiner (2006) proposed taxonomy of higher- and lower-order traits present in childhood and adolescence. The following criteria were used to determine the final list of characteristics included in the item pool: (a) the item must refer to an individual, internal characteristic; (b) consistent with a strengths-based approach, only positive characteristics were included; and (c) to reduce redundancy and create a measure of manageable length, adjectives that were similar in meaning were not included; rather, only one adjective that was most commonly found in the literature was included.

The characteristics included in the development of the PSI that were represented in both the parent focus groups and the developmental trait literature were: active, creative, curious, deliberate, empathic, enthusiastic, flexible, focused, generous, gregarious, helpful, humorous, leadership, open-minded, organized, persistent, positive, self-disciplined, and warm. Additional constructs drawn from the literature were: accepting, altruistic, calm, cooperative, goal-oriented, modest, and trustworthy.

Nine face-valid items were created for each characteristic (i.e., active, accepting, altruistic, calm, cooperative, creative, curious, deliberate, empathic, enthusiastic, flexible, focused, generous, goal-oriented, gregarious, helpful, humorous, leadership, modest, open-minded, organized, persistent, positive, self-disciplined, trustworthy, and warm), resulting in an initial pool of 234 items. Experts in developmental psychology (*n* = 1), early childhood education (*n* = 2), and counseling/clinical psychology (*n* = 3) reviewed the items for content validity and clarity. The experts included four women and two men; five of the experts identified as White and one identified as Latina. Reviewers were asked to examine (a) whether items were conceptually aligned with the strength/trait they were intended to assess and (b) structural features of the items such as clarity of wording, reading level required for comprehension, and ambiguity (DeVellis, 2017). The experts suggested clarifying the wording of the initial instructions, reducing the number of items presented at one time on the online survey screen, and using a progress bar on the online survey screen. The experts indicated they believed each item reflected the strength it was intended to measure and that the items were clear and concise. The suggestions provided by the experts were implemented, and the experts approved of the changes.

##### Response scale

The response format for the PSI was designed to address concerns about parents’ documented tendency to overestimate their children’s positive qualities (e.g., Kårstad et al., 2014; Lagattuta et al., 2012). The PSI response scale was modeled on the Perceived Self-Competence Scale (Harter, 1982), which was designed to limit social desirability related to perceived self-competence. With this format, for each item in the Perceived Self-Competence Scale, participants select which description from the two provided best reflects how they perceive themselves and then to what degree (i.e., “somewhat” or “very much”). Similarly, with the PSI, parents select the description that is most like their child (e.g., “Some children are typically pessimistic” or “Some children are typically optimistic”) and to what degree the description is like their child (i.e., “somewhat” or “very much”). Furthermore, the questions were written to describe children in third person and parents were asked to think about their child in the context of the questions; therefore, a sense of distance between the question and the child is established. Items on the PSI were scaled from 1 to 4, with higher scores indicating greater endorsement of the identified strength.

#### Participants and procedure

Parents/guardians (*N* = 302, 51% mothers, 49% fathers) of young children (ages 3–5) were recruited to participate from preschools and day care centers in the Midwestern United States (*n* = 119) and through the online data collection platform Qualtrics (*n* = 183). Participants from preschools and day care centers volunteered their time and did not receive any compensation for their participation. Participants recruited from Qualtrics received compensation in Qualtrics cash points ($4), which are exchanged for incentives the participants select. The sample was randomly divided into two groups, with equivalent representation of mothers and fathers in each group, to conduct exploratory and confirmatory factor analyses. This approach is similar to that used in other instrument development studies (e.g., Shoshani, 2019).

For the EFA subsample, participants’ mean age was 36.12 years (*SD* = 7.72) and consisted of 82% White, 6% Asian, 4% African American/Black, 4% Hispanic/Latino/a/x, 2% Multiracial, and 2% of another race/ethnicity. The parents reported the following level of education: 24.5% high school diploma/GED; 39.1% Bachelor’s degree; 22.5% Master’s degree; 7.3% M.D./Ph.D./J.D.; and 6.6% “other” education. Their children (49% girls; 51% boys) were 3 (33%), 4 (43%), and 5 (24%) years old. The children’s race/ethnicity were: 76% White, 10% Multiracial, 7% Asian, 3% African American/Black, 3% Hispanic/Latino/a/x, and 1% of another race/ethnicity.

For the CFA sample, participants’ mean age was 34.84 years (*SD* = 7.12). The parent sample consisted of 77% White, 7% Hispanic/Latino/a/x, 6% African American/Black, 5% Asian, 1% Multiracial, 1% of another race/ethnicity, and 2% did not disclose. The parents reported the following level of education: 0.7% less than high school diploma; 23.2% high school diploma/GED; 40.4% Bachelor’s degree; 17.9% Master’s degree; 7.3% M.D./Ph.D./J.D.; and 10.6% “other” education. Their children (56% girls, 44% boys) were ages 3 (35%), 4 (40%), and 5 (25%) years old. The children’s race/ethnicity were: 70% White, 9% Multiracial, 7% African American/Black, 7% Hispanic/Latino/a/x, 5% Asian, 1% of another race/ethnicity, and 1% not reported.

### Analytic strategy

Given that the underlying structure of early childhood strengths from a trait perspective was previously unknown, an EFA was selected as the first method of analysis. The EFA was conducted using the maximum likelihood method with a Promax (oblique) rotation. Several parameters were selected *a priori* given the sample size alongside data driven strategies to determine the number of factors included in the final model. Eigenvalues greater than one and the scree plot were initially examined; five or six factors were first identified. The factor loadings of the items were then evaluated. Given the sample size for the EFA was between 150 and 200, the recommended practice of retaining item loadings greater than or equal to 0.50 on at least one factor to ensure the item is a good measure of the overall factor (Tabachnick and Fidell, 2019; Worthington and Whittaker, 2006) and less than or equal to 0.25 on any other factor to eliminate high cross loadings was used. This method removed items that were either weakly loaded or cross-loaded on a number of factors. Finally, items were reviewed by the authors for weak internal validity and content. The final model resulted in a five-factor model consisting of 37 items (see Table 2).

**Table 2:** PSI items, EFA factor loadings, means, and standard deviations and CFA estimates, means, and standard deviations.

Factor/Item	EFA factor loadings					*M*	*SD*	CFA estimates	*M*	*SD*
Dynamic										
…are very outgoing	**0.83**	−0.07	−0.08	−0.01	0.11	3.21	0.97	0.45	3.13	0.95
…in an unfamiliar situation adapt well	**0.81**	0.11	−0.04	0.01	0.03	2.93	0.94	0.68	2.71	0.85
…find it easy to meet new people	**0.71**	−0.08	−0.08	−0.03	0.06	3.05	1.00	0.74	2.91	1.02
…are comfortable when plans change	**0.71**	0.21	0.03	−0.08	−0.03	2.73	0.97	0.44	2.65	0.95
…are ***frequently*** enthusiastic	**0.69**	−0.10	0.11	−0.00	−0.17	3.23	0.94	0.71	3.25	1.03
…are able to adapt to unfamiliar situations	**0.68**	0.09	−0.10	0.05	−0.01	2.75	1.02	0.77	2.69	0.92
…are eager to learn new things	**0.66**	0.04	0.06	0.18	0.00	3.38	0.89	0.57	3.40	0.81
…***frequently*** light up when talking with others	**0.61**	0.04	0.02	0.02	−0.03	2.98	1.12	0.73	3.09	1.07
…often come up with original ideas	**0.58**	0.05	0.07	0.20	−0.13	3.23	0.89	0.63	3.15	0.89
…are typically optimistic	**0.54**	−0.16	0.01	0.13	0.04	3.13	0.90	0.66	3.23	0.80
Dependable										
…are goal-orientated	−0.01	**0.92**	−0.08	0.07	0.06	2.96	0.77	0.86	2.75	0.76
…enjoy setting goals for themselves	0.05	**0.77**	0.13	−0.04	0.07	2.87	0.84	0.79	2.81	0.76
…***frequently*** work hard until they achieve their goal	−0.06	**0.68**	−0.11	0.03	0.04	2.86	0.90	0.62	2.66	0.91
…thrive on setting goals	0.01	**0.68**	0.08	−0.09	0.13	2.79	0.84	0.68	2.81	0.77
…can be trusted with sensitive information	0.03	**0.58**	0.05	−0.13	−0.01	2.75	0.88	0.60	2.60	0.83
…are ***consistently*** responsible	−0.07	**0.58**	0.12	0.02	−0.14	2.60	0.83	0.54	2.57	0.91
…can easily be depended on	0.11	**0.56**	0.24	−0.13	−0.11	3.13	0.80	0.67	3.07	0.79
…carefully plan their course of action	0.02	**0.50**	−0.16	0.12	−0.13	2.66	0.87	0.69	2.58	0.84
Caring										
…***frequently*** express compassion for those in pain	0.07	−0.19	**0.82**	−0.10	0.04	3.07	1.03	0.72	3.03	0.95
…are ***very*** helpful	0.02	0.05	**0.81**	0.10	−0.11	3.07	1.06	0.64	3.07	0.95
…are ***frequently*** empathic	−0.16	−0.05	**0.81**	−0.01	−0.04	3.03	0.94	0.74	2.99	0.96
…enjoy assisting their peers	0.02	0.05	**0.76**	0.01	0.17	3.19	0.88	0.76	3.17	0.84
…immediately assist others in need of help	−0.10	0.06	**0.72**	0.06	0.10	2.95	0.92	0.81	2.91	0.87
…***frequently*** help their peers and/or siblings	−0.12	0.13	**0.67**	0.09	0.04	3.05	0.97	0.72	2.96	0.99
…***frequently*** make gifts to give to family and friends	0.04	0.06	**0.67**	0.00	0.01	2.99	1.09	0.55	2.92	1.06
…can identify the emotions others are feeling	−0.01	0.06	**0.65**	0.04	−0.01	3.28	0.84	0.78	3.23	0.86
…are ***generally*** accepting of their peers, despite their differences	0.10	−0.14	**0.61**	−0.00	0.21	3.17	1.01	0.65	3.30	0.87
…are ***generally*** patient with others who have different ideas than they do	−0.08	−0.03	**0.57**	0.02	0.03	2.76	0.94	0.55	2.77	0.86
Organized										
…enjoy spending time organizing their possessions	−0.06	−0.08	0.01	**0.89**	−0.02	2.46	0.94	0.77	2.26	0.96
…enjoy organizing things	0.05	0.02	0.05	**0.88**	0.01	2.58	0.98	0.86	2.34	0.92
…like to arrange their toys	0.05	0.03	0.05	**0.86**	0.01	2.85	1.05	0.80	2.70	1.05
…enjoy categorizing their toys or books	0.03	−0.05	−0.05	**0.84**	0.02	2.83	0.98	0.90	2.58	1.02
Inspiring										
…often lead the group when playing	0.04	−0.04	0.04	−0.05	**0.90**	2.77	0.98	0.87	2.54	1.00
…are typically leaders	−0.01	0.10	−0.13	0.06	**0.82**	2.90	0.90	0.79	2.85	0.92
…tend to decide what the group will play	−0.00	0.01	0.07	−0.07	**0.77**	2.66	0.92	0.75	2.58	0.98
…***typically*** influence what the group will do	−0.06	−0.08	0.08	0.02	**0.72**	2.75	0.96	0.75	2.73	0.92
…***frequently*** direct the group	0.00	0.05	0.06	0.09	**0.72**	2.64	0.98	0.79	2.65	0.95

Note: Bold values represent factor loadings on the final primary factors.

Due to the ordered categorical nature of the items (i.e., dichotomous presentation), a robust weighted least squares estimator (WLSMV) was used to conduct the CFA (Muthén and Muthén, 2017). Four indices were used to evaluate the fit of the CFA model, including the comparative fit index (CFI), the Tucker-Lewis Index (TLI), the root mean square error of approximation (RMSEA), and the Chi Square of Model Fit (Chi Square). Hu and Bentler (1995) empirically examined a number of fit index cutoffs and suggested that in order to minimize Type I and Type II errors, a combination of an absolute fit index (e.g., RMSEA) and relative fit indices (e.g., CFI, TLI) should be used. The Chi-Square Test of Model Fit attempts to fit a model to the observed data, whereas the Chi-Square Test of Model Fit for the Baseline Model represents what the model is expected to be and serves as a null model (Muthén and Muthén, 2017). Therefore, the lower the chi-square value, the better the fit (Tabachnick and Fidell, 2019). As general guidelines, CFI and TLI values of 0.90 or above and RMSEA values of 0.06 or less are considered supportive of good model fit (Hu and Bentler, 1995). However, Browne and Cudeck (1993) caution model selection is subjective in nature and fit indices should not be used as a “mechanical decision process,” but rather as a tool to help guide the decision process (p. 157).

### Study 2 results

#### Exploratory factor analysis

For the final EFA model, the sample size was deemed acceptable given that each factor loading was 0.50 or higher with no high cross loadings (Guadagnoli and Velicer, 1988; Worthington and Whittaker, 2006). See Table 2 for factor loadings, means, and standard deviations. Overall, the five-factor model had strong factor loadings (0.50–0.92), did not have any substantial cross loadings (≤ 0.24), and could be identified by the “bend” in the scree plot.

##### Factors and factor interpretability

The items that remained following the implementation of the final decision rules for item retention aligned conceptually based upon trait theory. The five factors identified and corresponding percentage of variance accounted for by each factor included: *Dynamic* (21%)*, Dependable* (14%)*, Caring* (11%)*, Organized* (8%), and *Inspiring* (6%). A description of each factor follows. See Table 2 for all items organized by strength and Supplementary Appendix 1 for the full measure.

- *Dynamic* (10 items) describes a young child who is enthusiastic, positive, creative, flexible, curious, and gregarious.
- *Dependable* (8 items) describes a young child who is goal-oriented, deliberate, and trustworthy.
- *Caring* (10 items) describes a young child who is accepting of others, empathic, generous, and helpful.
- *Organized* (4 items) describes a young child who arranges, categorizes, and organizes.
- *Inspiring* (5 items) describes a young child who is decisive, directive, influential among a group, and a leader.

##### Reliability estimates

Internal consistency was examined for this sample. Cronbach’s alphas ranged from 0.82–0.89 across the 5 subscales, demonstrating good internal consistency. The reliability estimates for each subscale can be found in Table 3.

**Table 3:** Descriptive statistics and Cronbach’s alphas for the PSI’s five subscales across four samples by children’s age and gender.

	All Ages	3 year olds	4 year olds	5 year olds	Boys	Girls	Cronbach’s
	*M(SD)*	*M(SD)*	*M(SD)*	*M(SD)*	*M(SD)*	*M(SD)*	Alpha
EFA sample (*N* = 151)							
Dynamic	3.06(0.65)	3.16(0.50)	2.97(0.77)	3.09(0.59)	3.14(0.54)	2.98(0.74)	0.87
Dependable	2.83(0.56)	2.79(0.54)	2.90(0.56)	2.75(0.57)	2.81(0.55)	2.84(0.57)	0.82
Caring	3.06(0.68)	2.88(0.77)	3.17(0.60)	3.08(0.64)	2.95(0.70)	3.16(0.65)	0.89
Organized	2.68(0.86)	2.62(0.83)	2.66(0.93)	2.81(0.77)	2.70(0.81)	2.66(0.91)	0.89
Inspiring	2.74(0.77)	2.63(0.80)	2.86(0.75)	2.68(0.74)	2.61(0.75)	2.88(0.77)	0.87
CFA sample (*N* = 151)							
Dynamic	3.02(0.60)	3.07(0.44)	2.95(0.75)	3.06(0.50)	2.98(0.51)	3.05(0.65)	0.84
Dependable	2.73(0.55)	2.62(0.44)	2.88(0.62)	2.65(0.54)	2.66(0.53)	2.79(0.57)	0.83
Caring	3.04(0.62)	2.90(0.53)	3.20(0.62)	2.97(0.67)	2.87(0.57)	3.17(0.62)	0.86
Organized	2.47(0.83)	2.48(0.77)	2.50(0.88)	2.41(0.84)	2.42(0.84)	2.51(0.82)	0.86
Inspiring	2.67(0.76)	2.50(0.73)	2.88(0.78)	2.56(0.70)	2.64(0.76)	2.69(0.77)	0.86
Validity sample (*N* = 210)							
Dynamic	2.97(0.49)	3.01(0.50)	2.90(0.52)	3.02(0.43)	2.93(0.49)	3.01(0.49)	0.78
Dependable	2.57(0.53)	2.50(0.49)	2.59(0.51)	2.65(0.62)	2.54(0.54)	2.61(0.53)	0.83
Caring	3.07(0.50)	3.14(0.45)	2.95(0.52)	3.13(0.54)	2.99(0.53)	3.15(0.47)	0.78
Organized	2.73(0.83)	2.73(0.81)	2.83(0.85)	2.62(0.81)	2.78(0.90)	2.69(0.77)	0.89
Inspiring	2.64(0.69)	2.55(0.64)	2.56(0.70)	2.90(0.72)	2.62(0.66)	2.65(0.73)	0.86
Test–retest sample at time 2 (*N* = 98)							
Dynamic	2.98(0.48)	3.02(0.45)	2.97(0.50)	2.85(0.54)	3.02(0.51)	2.94(0.46)	0.78
Dependable	2.66(0.50)	2.69(0.45)	2.70(0.50)	2.49(0.62)	2.66(0.47)	2.66(0.52)	0.85
Caring	3.07(0.51)	3.15(0.50)	3.02(0.53)	2.99(0.52)	3.08(0.52)	3.07(0.51)	0.82
Organized	2.90(0.85)	2.85(0.87)	2.97(0.80)	2.88(0.95)	2.88(0.93)	2.92(0.79)	0.95
Inspiring	2.53(0.71)	2.53(0.64)	2.58(0.84)	2.43(0.55)	2.57(0.75)	2.51(0.67)	0.91

#### Confirmatory factor analysis

As noted previously, a separate sample was used to conduct the CFA. The five-factor model selected met or was just shy of the fit indices’ general guidelines (CFI = 0.90; TLI = 0.89; RMSEA = 0.06), including the Chi-Square Test of Model Fit value (905.64, *p* < 0.001), which was less than the Chi-Square Test of Model Fit for the Baseline Model (3404.09, *p* < 0.001). Together, the five-factor model selected was deemed adequate (Browne and Cudeck, 1993). The estimates and descriptives from the CFA can be found in Table 2.

##### Reliability estimates

Internal consistency was also examined for this sample. Cronbach’s alphas ranged from 0.83–0.86 across the five subscales, demonstrating good internal consistency (see Table 3).

## Study 3: validity and test–retest reliability

The purpose of study 3 was to examine reliability and validity of the PSI. To do so, the PSI was administered twice, 1 month apart, and existing, validated measures of personality traits and social skills were administered to a sample of parents of young children.

### Study 3 methods

#### Participants

A sample of 210 parents/guardians of young children (ages 3–5) from across the United States (50% mothers, 50% fathers) was recruited via Qualtrics. Participants received compensation for their participation in Qualtrics cash points ($4). The sample had a mean age of 33.31 years (*SD* = 5.26) and consisted of 82% White, 8% Hispanic/Latino/a/x, 5% African American/Black, 2% Asian, 2% Multiracial, and 1% Native American. The parents reported the following level of education: 0.5% less than high school diploma; 30.5% high school diploma/GED; 36.7% Bachelor’s degree; 17.1% Master’s degree; 4.8% M.D./Ph.D./J.D.; 9.5% “other” education; and 1% did not report. Family income levels were reported as: 2.9% with the range of $10,000–$14,000; 7.1% with the range of $15,000–$24,999; 6.7% with the range of $25,000–$34,999; 15.2% with the range of $35,000–$49,999; 25.2% with the range of $50,000–$74,999; 20.5% with the range of $75,000–$99,999; 21.9% with the range of $100,000 and above; and 0.5% did not report. Their children (51% girls; 49% boys) were ages 3 (39%), 4 (37%), and 5 (23%) years old; 1% did not report their child’s age. The children’s race/ethnicity were: 79% White, 9% Hispanic/Latino/a/x, 6% Multiracial, 3% African American/Black, 2% Asian, and 1% Native American.

From the Study 3 sample, 98 parents/guardians participated in the PSI test–retest administration by completing the PSI a second time 1 month later. These participants also received compensation for their participation in Qualtrics cash points ($4). Of the test–retest subset of the sample, 69.7% were mothers and 29.3% were fathers. The mean age was 34.10 years (SD = 5.02) and 87.9% were White, 6.1% Hispanic/Latino/a/x, 1% African American/Black, 1% Asian, 2% Multiracial, and 1% Native American. The parents reported the following level of education: 29.2% high school diploma/GED; 33.3% Bachelor’s degree; 23.2% Master’s degree; 5.1% M.D./Ph.D./J.D.; 8.1% “other” education; and 1% did not report. Family income levels were reported as: 1.0% with the range of $10,000–$14,000; 4.0% with the range of $15,000–$24,999; 5.1% with the range of $25,000–$34,999; 16.2% with the range of $35,000–$49,999; 24.2% with the range of $50,000–$74,999; 19.2% with the range of $75,000–$99,999; 29.3% with the range of $100,000 and above; and 1% did not report. Their children (54.5% girls; 44.4% boys) were ages 3 (46.5%), 4 (37.4%), and 5 (15.2%) years old; 1% did not report their child’s age. The children’s race/ethnicity were: 82.8% White, 6.1% Hispanic/Latino/a/x, 7.1% Multiracial, 1% African American/Black, and 2% Asian; 1% did not report their child’s race/ethnicity.

#### Measures and procedure

Scores from the five subscales of the PSI were compared to existing, validated measures of two related constructs: personality traits and social skills. Related, yet distinct, constructs were of interest to examine validity. Specifically, personality traits and social competence were selected given the identification of interpersonal and intrapersonal strengths in the PSI. Moreover, given the ongoing discussion of the similarities and differences between strengths and personality traits in existing research literature (e.g., Dametto and Noronha, 2021; McGrath et al., 2020; Najderska and Cieciuch, 2018; Ruch et al., 2023), examining their relations was also of particular interest. It was expected that the five factors identified by the PSI would correlate with the personality traits of openness, conscientiousness, extraversion, agreeableness, and neuroticism in construct-specific ways. For example, it was expected that the *Dynamic* subscale would be positively correlated with openness and extraversion, the *Dependable* and *Organized* subscales would be positively correlated with conscientiousness, and the *Caring* subscale would be positively correlated with agreeableness. It was expected that the PSI factors involving more interpersonal characteristics (e.g., *Caring*) would positively correlate with social skills.

##### Personality traits

Personality traits were measured using the Big Five Inventory (BFI; John and Srivastava, 1999). The parent report version of the BFI is a 44-item measure that assesses parents’ perceptions of their children across the Big Five Factors of personality (John et al., 1991; John et al., 2008). It includes five subscales: Openness, Conscientiousness, Extraversion, Agreeableness, and Neuroticism. The average test–retest reliability was 0.83 across 8 weeks in an English sample and 0.85 across 6 weeks in a German sample (Rammstedt and John, 2007). On average, in past studies, the convergent validity correlations between the BFI and the NEO-PI-R was 0.78. For this study’s sample, internal consistencies were 0.83 for the Extraversion subscale, 0.78 for the Agreeableness subscale, 0.86 for the Conscientiousness subscale, 0.83 for the Neuroticism subscale, and 0.74 for the Openness subscale.

##### Social skills

Social skills were measured using the Social Competence Scale—Parent Version (SCS; Corrigan, 1995). The SCS is a 12-item measure with two subscales: Prosocial/Communication Skills and Emotional Regulation Skills. The SCS was originally designed for elementary-school-aged children but has been validated with a preschool-aged sample (Gouley et al., 2008). With the past preschool-aged sample, internal consistency ranged from 0.87 to 0.92. The SCS demonstrated concurrent validity with the Social Skills Rating Scale—Preschool Version (Gresham and Elliott, 1990), Emotion Regulation Checklist (Shields and Cicchetti, 1997), Penn Interactive Peer Play Scale (Fantuzzo et al., 1995), New York Rating Scale (Miller et al., 1995), Child Behavior Checklist (Achenbach, 1999), the difficult child subscale from the Parenting Stress Index—Short Form (Abidin, 1995), and cognitive ability using the Differential Abilities Scale (Elliott, 1990). For this study’s sample, the internal consistencies were 0.80 for the Prosocial/Communication Skills subscale and 0.81 for the Emotional Regulation Skills subscale.

### Study 3 results

#### Validity

Regarding validity, the results of the correlational analysis between the subscales of the PSI, BFI, and the SCS are included in Table 4. Correlations ranged from −0.60 to 0.68, and relations between variables were generally in the expected directions and comparable in size to relations in the literature (e.g., Asendorpf and Van Aken, 2002; Barbaranelli et al., 2003; John and Gross, 2007; Purnamaningsih, 2017). For example, the *Dynamic* subscale of the PSI was positively correlated with the openness and extraversion subscales of the BFI, *r* = 0.36, *p* < 0.001 and *r* = 0.68, *p* < 0.001, respectively. The *Dependable* and *Organized* subscales of the PSI were positively correlated with the conscientiousness subscale of the BFI, *r* = 0.65, *p* < 0.001 and *r* = 0.47, *p* < 0.001, respectively. The *Caring* subscale of the PSI was positively correlated with the agreeableness subscale of the BFI, *r* = 0.54, *p* < 0.001, as well as the prosocial/communication skills and emotion regulation skills subscales of the SCS, *r* = 0.63, *p* < 0.001 and *r* = 0.37, *p* < 0.001, respectively.

**Table 4:** Correlations between the PSI and other measures (*N* = 210).

Measure SUBSCALE	1	2	3	4	5	6	7	8	9	10	11	12
PSI Dynamic	–	0.24**	0.40**	−0.15*	0.09	0.36**	0.05	0.68**	0.50**	−0.51**	0.25**	0.10
PSI Dependable		–	0.39**	0.22**	0.11	0.24**	0.65**	0.04	0.33**	−0.28**	0.45**	0.47**
PSI Caring			–	0.08	0.10	0.29**	0.34**	0.34**	0.54**	−0.34**	0.63**	0.37**
PSI Organized				–	0.14*	0.08	0.47**	−0.05	0.02	−0.04	0.16*	0.18**
PSI Inspiring					–	0.28**	0.12	0.32**	−0.11	−0.03	0.03	0.06
BFI openness						–	0.25**	0.41**	0.27**	−0.20**	0.29**	0.10
BFI conscientiousness							–	0.02	0.42**	−0.36**	0.51**	0.59**
BFI extraversion								–	0.33**	−0.34**	0.19**	−0.13
BFI agreeableness									–	−0.60**	0.57**	0.43**
BFI neuroticism										–	−0.41**	−0.47**
SCS prosocial/communication skills											–	0.67**
SCS emotional regulation skills												–

PSI, Preschool Strengths Inventory; BFI, Big Five Inventory; SCS, Social Competence Scale. **p < 0.01 (two-tailed), *p < 0.05 (two-tailed).

Results also indicated areas of both similarity and distinctiveness across the PSI subscales. For example, both *Dynamic* and *Inspiring* were positively correlated with the personality traits of extraversion and openness, but the relations were stronger for *Dynamic* than for *Inspiring*. *Dynamic* was also positively correlated with agreeableness and negatively correlated with neuroticism, whereas *Inspiring* was unrelated to these two constructs. These results are consistent with the definition of *Dynamic* as focused on positive interpersonal relationships, whereas *Inspiring* is more focused on interpersonal leadership.

Together, these results suggest that strengths represent the positive end of trait continua and are related to expected, similar traits and interpersonal skills. However, the significant correlations previously described were generally moderate in nature. This suggests that although the variables demonstrate some overlap in features, they are discrete variables.

#### Test–retest reliability

Pearson correlations across each subscale ranged from 0.78–0.88 for a one-month period. The individual subscales were 0.81 (*Dynamic*), 0.85 (*Dependable*), 0.86 (*Caring*), 0.78 (*Inspiring*), and 0.88 (*Organized*). Results suggest good consistency over time.

## General discussion

It is apparent from past research that there are many benefits to identifying and fostering strengths across contexts (Ghielen et al., 2018; Lavy, 2019). However, less attention has been given to children, particularly young children, in the strengths literature. This gap is concerning, as young children are at a prime age to acquire positive messages about the self that could promote success and allow them to thrive in the future (Cabaj et al., 2014; Orth, 2018). In addition, although strengths are thought to be relatively stable across the lifespan (Bowers et al., 2010; Owens et al., 2018), a lack of research on strengths in childhood contributes to gaps in understanding of when such stability might emerge. Thus, the aim of this study was to develop a brief, reliable, and valid measure to identify preschool-aged children’s strengths. Such a tool could help build the strengths literature for young children and provide a means to foster young children’s strengths early in life.

The results presented in this paper provide support for the content structure, reliability, and validity of the PSI. Results from the EFA indicated that a five-factor model best described the data. The broad factors identified were *Dynamic* (i.e., enthusiastic, positive, creative, flexible, curious, gregarious), *Dependable* (i.e., goal-oriented, deliberate, trustworthy), *Caring* (i.e., accepting, empathic, generous, helpful)*, Inspiring* (i.e., decisive, directive, influential, shows leadership), and *Organized* (i.e., children who arrange, categorize, and organize things). The five-factor model identified by the EFA was confirmed by a CFA. Additionally, the psychometrics of the measure, including internal consistency, test–retest reliability over 1 month, and relations with measures of personality traits and social skills, were good.

The primary structure of the PSI was formed based on data from parent focus groups, with additional input from extant research on childhood temperament, personality, and traits. Although the goal of developing the PSI measure was not to measure constructs highlighted within extant developmental research, it is important to note similarities between the strengths identified in the PSI and characteristics identified in existing theoretical and empirical literature. First, a variety of elements of the PSI aligned with the positive end of the trait continuum of the five-factor personality model identified in adolescents and adults. For example, traits under the PSI factors *Dependable* (goal-oriented, deliberate) and *Organized* (organizes) parallel lower-order traits typically subsumed by the higher-order factor of conscientiousness (self-control, achievement motivation, orderliness; Caspi and Shiner, 2006). Similarly, traits under the PSI factor *Caring* (helpful, empathic) parallel the lower-order traits typically under the higher-order factor agreeableness (prosocial tendencies; Caspi and Shiner, 2006). Traits within the PSI factor *Dynamic* (enthusiastic, gregarious, creative, curious) overlap with lower-order traits of both extraversion (sociability, energy level) and openness to experience (creativity, curiosity; Caspi and Shiner, 2006).

Although the elements identified by the PSI show some overlap with existing constructs, there are also meaningful distinctions and contributions of the measure as a whole. First, as mentioned previously, strengths are intended to measure positive characteristics that are believed to promote optimal functioning (Biswas-Diener et al., 2011; Louis and Lopez, 2014; Owens et al., 2018). Therefore, only positively valenced characteristics were included in the development of the PSI. Second, the traits identified in the development process and their organization within the broad factors of the PSI are not identical to the five factor model of personality. For example, the *Dynamic* construct shows some overlap with the sociability and positive affect elements of many theories and measures of early childhood temperament, but also includes other constructs such as curiosity and creativity. Elements of the *Dependable* and *Organized* factors align with elements of conscientiousness, but *Dependable* and *Organized* are each distinct PSI factors that are comprised of additional unique characteristics (e.g., to beginning of parenthetical statement trustworthy, arranges). The *Inspiring* factor does not parallel a specific domain of the five factor model of personality. Third, the *Caring* factor has some overlap with social competence; however, it also involves other related, but discrete characteristics, such as acceptance of others. These findings were reflected in the validity study. The constructs measured (i.e. personality and social skills) were generally correlated with the PSI factors in the expected directions to a moderate degree, suggesting they are related, yet distinct from one another. This provides further support for the claim that strengths and personality traits are unique and also suggests social skills are distinct from interpersonal strengths. Finally, strengths may also influence how young children engage with developmental tasks (Mahatmya et al., 2012). For example, *Caring* and *Inspiring* may reflect different approaches to the developmental task of engaging in peer play, whereas *Dynamic* and *Dependable* may reflect strategies for succeeding in preschool environments that reward goal-oriented behavior, intellectual curiosity, and flexibility in adapting to new situations.

While one strengths measure has been previously developed for young children—the CSI-EC (Shoshani, 2019)—the PSI addresses some of its limitations and offers a different measure with distinct strengths of its own. With the CSI-EC, the 24 character strengths that are part of the VIA character strengths model—initially developed for and with adults—were generalized to a young child sample. As noted previously, in developing the PSI, the researchers utilized a bottom-up approach in which parent focus groups were conducted to identify strengths present in young children and developmental literature was later reviewed to be as thorough as possible. This research approach resulted in different traits and factors being identified than the CSI-EC. The CSI-EC interpersonal strengths of kindness and love would likely be similar to traits encompassed by the *Caring* factor in the PSI (e.g., generous, helpful). The CSI-EC interpersonal strength of leadership aligns with some of the traits present within the PSI *Inspiring* factor (e.g., leader). The CSI-EC intellectual strengths of curiosity and creativity appear similar to the traits within the PSI *Dynamic* factor (i.e., creative, curious). The CSI-EC transcendence strength of zest parallels the trait of enthusiasm present in the *Dynamic* factor of the PSI. The remaining traits represented in the PSI (listed previously in the Factors and Factor Interpretability section; e.g., positive, flexible, goal-oriented, organized, decisive) appear distinct from the strengths in the CSI-EC. Finally, the PSI was also designed to be brief in nature, consisting of 37 items; the CSI-EC has 97 items. A brief measure will hopefully allow parents, practitioners, teachers, and researchers alike to assess young children’s strengths efficiently. Thus, although there is some overlap in the strengths represented in the CSI-EC and PSI, the differences present in the PSI and the approach in which the PSI was developed offer a unique contribution.

### Implications

Although strengths are generally stable across time, external factors, such as role models, experiences, and interventions, can influence the overall impact of strengths (e.g., Biswas-Diener et al., 2011; Ghielen et al., 2018; Owens et al., 2018). Strengths interventions are designed to promote awareness of strengths and encourage individuals to find ways to use and strengthen their existing strengths (Ghielen et al., 2018; Louis and Lopez, 2014; Quinlan et al., 2012). Often, the first step in such a process is to identify the strengths present within the person (Clifton et al., 2006). Everyone has their own unique constellation of strengths, with no set of strengths being better or worse than another (Clifton et al., 2006; Peterson and Seligman, 2004). The PSI will help address this important first step by systematically establishing what strengths are present in young children. Once identified, these strengths can be used to promote well-being through activities, programs, and interventions in a variety of contexts, such as home, school, therapy, and recreational programs. For example, awareness of student strengths and the ability to assess them easily could help early childhood educators to provide the kind of personalized, student-centered instruction that is most beneficial for students (Darling-Hammond et al., 2023). Awareness of individual strengths could also help to promote a better understanding of well-being and strategies for promoting well-being among young children (Lottman et al., 2017; Waters et al., 2022).

A recent review of positive psychological interventions (PPIs) for children and adolescents highlighted the scarcity of interventions and programs that focus specifically on strengths, particularly for early childhood populations and settings (Owens and Waters, 2020). With the PSI, greater attention to strengths in young children may be possible, furthering the potential for strengths-based preventative and intervention efforts, and subsequent beneficial outcomes (e.g., enhanced well-being, decreased mental health concerns) early in life. Future research could also use the PSI to assess the impact of interventions; for example, would executive functioning interventions lead to increases in the dimensions of *Dependable* or *Organized*?

The creation of the PSI will also help further research endeavors. As noted previously, the PSI could be used to identify strengths that are the focus of strength development programs and interventions. The efficacy of such interventions could then be assessed through randomized clinical trials. By doing so, evidence-based approaches to developing strengths could be established and later used in applied contexts. Strengths identified from the PSI can also be examined in relation to other outcomes or mediating and moderating variables of interest in cross-sectional and longitudinal research. In addition, the developmental trajectory of strengths can be examined from a trait perspective starting early in life. Together, the PSI provides a tool to help further the research base in young children’s strengths.

### Limitations and future directions

A few limitations and future directions are important to consider. One limitation is that the EFA and CFA analyses had a less than ideal sample size. Given this consideration, steps were taken to address this or limit its impact. First, fairly equivalent samples of mothers and fathers across the United States were intentionally sought during recruitment to increase the generalizability and applicability of the PSI. Second, when conducting the EFA, *a priori* decision rules—following best practice guidelines—were used so that the items selected for retention were appropriate for the sample size; items with factor loadings under 0.50 or with cross-loading at or above 0.25 were excluded. Concerns regarding the sample size were also reduced when the factor structure from the EFA was supported by the CFA.

Relatedly, while gender (mothers and fathers for all samples), education levels (for all samples), and family income levels (for samples 2 and 3) were fairly well distributed, another limitation across all samples was the limited diversity related to race and ethnicity. Future research could include additional validity studies with the goal of expanding the diversity of the parents and children represented, particularly related to race and ethnicity. Additionally, examining the factor structure of the PSI with different cultural groups would determine whether or not the strengths identified in the current measure are applicable to other communities and what adaptations are needed.

Finally, the current study relied on parents to report their perceptions of their children’s strengths. Although parents are a valid source of data about young children, parents’ views of what is a strength in early childhood may differ from the views of other important adults (such as early childhood educators) or of children themselves. Thus, creating a teacher version of the PSI for use by early childhood educators would provide another important perspective on young children’s strengths, as children may display different strengths in home and school contexts. Similarly, asking children to report on their own self-perceived strengths could help to better foster the aspects of the self that children are most interested in and passionate about (Galloway and Reynolds 2015).

## Conclusion

With research on young children’s strengths in its infancy, from both a research and practice standpoint, the PSI fills a gap in the literature. It provides a brief measure to systematically identify young children’s strengths. The use of this instrument could be helpful in developing evidence-based strengths programs and interventions as well as examining strengths longitudinally over time, which holds the potential to enhance young children’s lives.

## Data Availability

The data that support the findings of this article can be made available to qualified individuals on reasonable request from the corresponding author.
