# Supplementary material for: The preschool strengths inventory: development and validation
Source: Front Psychol. 2025 Feb 12;16:1468944. doi: 10.3389/fpsyg.2025.1468944 (PMC11860962; doi:10.3389/fpsyg.2025.1468944)
Supplement: Supplementary file 1 [file Data_Sheet_1.docx]

**Preschool Strengths Inventory**

Below are some statements that can describe children. We are interested in which choice best describes *your* child. If you have more than one child between the ages of 3 and 5, please complete the question thinking of *one* child. You can complete another survey and answer questions about your other child/children.

Sometimes you may find it hard to decide between the two choices; however, please answer each item. Once you decide which description is most like your child, please mark if you believe it is “really true” for your child or “sort of true.” You will only mark one box for each item. There are no right or wrong answers and every child is unique, so please be honest in your answers.

Here is a sample question.

|  | Really True for my Child | Sort of True for my Child |  |  |  | Sort of True for my Child | Really True for my Child |
| --- | --- | --- | --- | --- | --- | --- | --- |
| 0. |  |  | Some children prefer to play inside. | **BUT** | Some children prefer to play outside. |  |  |

In this case, first decide whether you believe your child would prefer to play inside or if they would prefer to play outside. Once you decide between the two options, then decide if it is “really true” for your child or “sort of true” for your child.

For each of the items below, decide which description best describes your child and mark whether it is “really true” or “sort of true” for your child.

|  | Really True for my Child | Sort of True for my Child |  |  |  | Sort of True for my Child | Really True for my Child |
| --- | --- | --- | --- | --- | --- | --- | --- |
| 1. |  |  | Some children are typically pessimistic. | **BUT** | Some children are typically optimistic. |  |  |
| 2. |  |  | Some children do not express much interest in learning new things. | **BUT** | Some children are eager to learn new things. |  |  |
| 3. |  |  | Some children are introverted. | **BUT** | Some children are very outgoing. |  |  |
| 4. |  |  | Some children are typically leaders. | **BUT** | Some children are typically followers. |  |  |
| 5. |  |  | Some children are ***occasionally*** enthusiastic. | **BUT** | Some children are ***frequently*** enthusiastic. |  |  |
| 6. |  |  | Some children have difficulty adapting to unfamiliar situations. | **BUT** | Some children are able to adapt to unfamiliar situations. |  |  |
| 7. |  |  | Some children shy away from setting goals. | **BUT** | Some children thrive on setting goals. |  |  |
| 8. |  |  | Some children ***cannot*** easily be depended on. | **BUT** | Some children can easily be depended on. |  |  |
| 9. |  |  | Some children often lead the group when playing. | **BUT** | Some children often go along with what the group is playing. |  |  |
| 10. |  |  | Some children do ***not*** enjoy setting goals for themselves. | **BUT** | Some children enjoy setting goals for themselves. |  |  |
| 11. |  |  | Some children like to arrange their toys. | **BUT** | Some children do ***not*** enjoy arranging their toys. |  |  |
| 12. |  |  | Some children can identify the emotions others are feeling. | **BUT** | Some children struggle to identify the emotions others are feeling. |  |  |
| 13. |  |  | Some children are ***occasionally*** responsible. | **BUT** | Some children are ***consistently*** responsible. |  |  |
| 14. |  |  | Some children have difficulty coming up with original ideas. | **BUT** | Some children often come up with original ideas. |  |  |
| 15. |  |  | Some children are ***not*** goal-orientated. | **BUT** | Some children are goal-orientated. |  |  |
| 16. |  |  | Some children enjoy categorizing their toys or books. | **BUT** | Some children ***dislike*** categorizing their toys or books. |  |  |
| 17. |  |  | Some children tend to decide what the group will play. | **BUT** | Some children tend to follow what the group is playing. |  |  |
| 18. |  |  | Some children have difficulty meeting new people. | **BUT** | Some children find it easy to meet new people. |  |  |
| 19. |  |  | Some children do ***not*** plan their course of action. | **BUT** | Some children carefully plan their course of action. |  |  |
| 20. |  |  | Some children ***frequently*** help their peers and/or siblings. | **BUT** | Some children ***occasionally*** help their peers and/or siblings. |  |  |
| 21. |  |  | Some children struggle when plans change. | **BUT** | Some children are comfortable when plans change. |  |  |
| 22. |  |  | Some children enjoy spending time organizing their possessions. | **BUT** | Some children do ***not*** care to spend time organizing their possessions. |  |  |
| 23. |  |  | Some children immediately assist others in need of help. | **BUT** | Some children may shy away from helping others in need. |  |  |
| 24. |  |  | Some children ***occasionally*** work hard until they achieve their goal. | **BUT** | Some children ***frequently*** work hard until they achieve their goal. |  |  |
| 25. |  |  | Some children are ***generally*** accepting of their peers, despite their differences. | **BUT** | Some children are ***occasionally*** accepting of their peers, despite their differences. |  |  |
| 26. |  |  | Some children enjoy assisting their peers. | **BUT** | Some children find it less enjoyable to assist their peers. |  |  |
| 27. |  |  | Some children are more difficult to trust with sensitive information. | **BUT** | Some children can be trusted with sensitive information. |  |  |
| 28. |  |  | Some children ***frequently*** direct the group. | **BUT** | Some children ***occasionally*** direct the group. |  |  |
| 29. |  |  | Some children in an unfamiliar situation have trouble adapting. | **BUT** | Some children in an unfamiliar situation adapt well. |  |  |
| 30. |  |  | Some children are ***generally*** patient with others who have different ideas than they do. | **BUT** | Some children are ***occasionally*** patient with others who have different ideas than they do. |  |  |
| 31. |  |  | Some children are ***frequently*** empathic. | **BUT** | Some children are ***occasionally*** empathic. |  |  |
| 32. |  |  | Some children ***frequently*** make gifts to give to family and friends. | **BUT** | Some children ***occasionally*** make gifts to give to family and friends. |  |  |
| 33. |  |  | Some children ***typically*** influence what the group will do. | **BUT** | Some children ***occasionally*** influence what the group will do. |  |  |
| 34. |  |  | Some children ***occasionally*** light up when talking with others. | **BUT** | Some children ***frequently*** light up when talking with others. |  |  |
| 35. |  |  | Some children enjoy organizing things. | **BUT** | Some children ***don’t*** particularly enjoy organizing things. |  |  |
| 36. |  |  | Some children ***frequently*** express compassion for those in pain. | **BUT** | Some children ***occasionally*** express compassion for those in pain. |  |  |
| 37. |  |  | Some children are ***very*** helpful. | **BUT** | Some children are ***occasionally*** helpful. |  |  |

**Scoring:**

From left to right, score 1-4 points.

The following items need to be reversed scored: 4, 9, 17, 28, 33 (all Inspiring items); 11, 16, 22, 35 (all Organized items); 12, 20, 23, 25, 26, 30, 31, 32, 36, 37 (all Caring items).

Calculate the average for each factor.

**Factors & Corresponding Items:**

*Dynamic*

1, 2, 3, 5, 6, 14, 18, 21, 29, 34

*Dependable*

7, 8, 10, 13, 15, 19, 24, 27

*Caring*

12, 20, 23, 25, 26, 30, 31, 32, 36, 37

*Inspiring*

4, 9, 17, 28, 33

*Organized*

11, 16, 22, 35
